# Treatment Resistance: A Time-Based Approach for Early Identification in First Episode Psychosis

**DOI:** 10.3390/jpm11080711

**Published:** 2021-07-24

**Authors:** Kara Dempster, Annie Li, Priyadharshini Sabesan, Ross Norman, Lena Palaniyappan

**Affiliations:** 1 Department of Psychiatry, Dalhousie University, Halifax, NS B3H 2E2, Canada; Kara.Dempster@nshealth.ca; 2 Department of Anaesthesia, Schulich School of Medicine & Dentistry, Western University, London, ON N6A 5B7, Canada; Annie.Li@lhsc.on.ca; 3 Department of Psychiatry, Western University, London, ON N6A 5B7, Canada; Priyadharshini.Sabesan@lhsc.on.ca (P.S.); rnorman@uwo.ca (R.N.); 4 Lawson Health Research Institute, London, ON N6C 2R5, Canada; 5 Robarts Research Institute, Western University, London, ON N6A 5B7, Canada

**Keywords:** treatment resistant schizophrenia, first episode psychosis, treatment response, first episode schizophrenia, early intervention

## Abstract

Although approximately 1/3 of individuals with schizophrenia are Treatment Resistant (TR), identifying these subjects prospectively remains challenging. The Treatment Response and Resistance in Psychosis working group defines <20% improvement as an indicator of TR, though its utility in First Episode Schizophrenia (FES) remains unknown. In a prospective cohort of FES (*n* = 129) followed up for 5 years, we evaluated two improvement thresholds for ‘probable TR’; <20% and <50% based on positive, negative, and total symptoms. We ascertained (1) the ecological validity (i.e., the ability to identify an expected subgroup of 1/3rd of patients); (2) the predictive validity (i.e., ability to predict poor global functioning) and (3) the clinical utility (association with clozapine use at the 5th year). Using the criteria of a total symptom reduction of <50% or negative symptom reduction of <20% resulted in ‘probable TR’ rates of 37% and 33%, respectively. Using <20% positive or total symptoms criteria resulted in very low rates, indicating minimal utility in FES. <50% total symptom criterion best predicted the global functioning over 5 years. Clozapine use was only predicted by positive symptom criterion. Prospective characterization of TRS is possible at 6 months after FES through a time-based approach using a 50% threshold for symptom change in treatment-adherent patients.

## 1. Introduction

Approximately one third of patients with schizophrenia [1,2] continue to experience symptoms despite treatment with dopamine-blocking antipsychotic agents. Treatment resistance (TR) occurs early in the illness trajectory [3], though clinical identification is often delayed [4]. TR has been primarily defined on the basis of measuring positive symptom response to sequential antipsychotic trials, with most cases identified retrospectively in patients with chronic schizophrenia [5]. A large body of evidence supports the existence of a sizeable subgroup of patients who are unlikely to respond optimally to first-line dopaminergic treatments, and are identifiable in first episode clinics through chart reviews (Demjaha et al., 2017; Demjaha et al., 2014) [6,7]. Lally and colleagues traced the records of 246 patients with FES from a single site over 5 years and estimated 33% to meet the criteria for TR; of these, only 50% were commenced on clozapine [8].

While the sequential failure of 2 adequately dosed antipsychotics is used to define TR, in practice, more than 4 antipsychotics are tried often in higher doses and in combinations, as practitioners fail to suspect TR earlier in the illness course [7]. To facilitate early identification of TRS, the Treatment Response and Resistance in Psychosis (TRRIP; [5]) working group published consensus guidelines defining treatment resistance. According to these guidelines, insufficient response is defined as <20% improvement on positive or negative symptom domains after 6 weeks of adhering to therapeutic dose for each of 2 different antipsychotics. However, implementing these recommendations for early identification of TR in FES is challenging for various reasons; (1) In FES samples, the response rates for positive symptoms are generally high [9,10]; it is unclear whether the threshold of <20% symptom improvement will be appropriate to identify the TRS subgroup (2) the value of measuring negative symptoms when defining TR in FES has not yet been characterized, as positive symptoms continue to assume a central role when evaluating treatment response [11], especially in early stages of psychosis (3) The recommended use of lowest possible effective doses and higher rates of apprehension for side effects when treating FES [12] prolongs treatment duration with a single agent further than 6 weeks in most cases (4) Oral medications are the most common route of administration in FES clinics [13]; but adherence is rarely measured on a routine basis. As a result, a determination of TR in FES takes much longer than the consensus recommendations, with a 4 to 9.7 years lag between a diagnosis of FES and the start of clozapine [14,15]. Substantial disability accumulates during this time, with the delay eventually reducing the probability of responding to clozapine as well [16,17,18].

Given the practical challenges in adopting conventional ‘trial-based’ TRS criteria in FES clinics, we study the utility of applying a “time-based” response cut-off, irrespective of the number of antipsychotic trials in one of the few FES samples in the world that was prospectively followed up for 5 years. We had two specific aims:

(1) To identify the symptom domains and thresholds that define a “probable TR” subgroup in a prospective manner in FES samples as early as 6 months after presentation. We studied the utility of a 20% response threshold (as defined by TRIPP; [5]) as well as a more stringent 50% response threshold (identified as a “good response” cut-off for clinical trials by Aboraya et al., (2017) [19] in the domains of positive, negative, and total symptoms 6 months following FES. In keeping with previous literature [1,2], and the single-site data from a 5-year follow-up of FES, we hypothesized that the most valid criteria would categorize approximately 33% of the sample as probable TR.
(2) To test the predictive and clinical validity of the various “probable TR” definitions at 6 months by assessing whether global functioning over 5 years and clozapine use at the 5th year could be reliably predicted on the basis of these definitions. Given the high response rates for positive symptoms expected in FES, we hypothesized that the use of 50% threshold as well as the inclusion of negative symptoms would be important in characterizing probable TR in a FES sample. Nevertheless, given the historical focus on positive symptoms when prescribing clozapine [11], we expected probable-TR defined as per positive rather than negative symptom thresholds to relate to eventual clozapine use.

## 2. Materials and Methods

### 2.1. Sample Recruitment

Data were analysed retrospectively using a longitudinal, naturalistic sample of 129 patients treated at the Prevention and Early Intervention Program for Psychosis (PEPP) in London, Ontario between February 1997 and February 2002. This program provides assessment and treatment to individuals presenting with first-episode, non-affective psychoses using an assertive case-management model. Criteria for acceptance to the program include age between 16 and 50, symptoms meeting criteria for a Diagnostic and Statistical Manual of Mental Disorders (Fourth Edition) (DSM-IV; APA, 2000) psychotic disorder, and having never received prior antipsychotic treatment for greater than one month. All patients were treated within the same program. Approval for the study was obtained from the University Human Ethics Committee for Health Sciences at the University of Western Ontario. Written informed consent was obtained from all subjects involved in the study.

### 2.2. Clinical Assessment

Diagnoses were established using the Structured Clinical Interview for DSM-IV (First & Gibbon, 1997) by trained research assistants, and confirmed by two senior psychiatrists and a clinical research psychologist, with consensus diagnosis conferences occurring at one-year follow-up. Individuals that ended up meeting criteria for a mood disorder with psychotic features, or any substance induced psychosis, were excluded from the analysis. Positive and negative symptoms of psychosis were assessed using the SANS (Andreasen, 1983) and SAPS (Andreasen, 1984) at baseline, and at months 1, 2, 3, and 6. Interrater reliability on the SAPS and SANS demonstrated agreement within one point 93% of the time [20]. Total symptoms were defined as the total score of the SAPS and SANS. Duration of untreated illness (DUI) was calculated as the period between the onset of any psychiatric symptoms and the time to antipsychotic treatment. Duration of untreated psychosis (DUP) was defined as the time between the onset of psychotic symptoms and the time to adequate antipsychotic treatment. Premorbid adjustment was measured using the Premorbid Adjustment Scale (PAS; [21]), with higher values indicating worse premorbid functioning. Symptom change overtime was calculated as the difference between a particular symptom domain from baseline divided by the baseline symptom score (eg positive symptom improvement over month 1 = (SAPS baseline-SAPS month 1)/SAPS baseline)).

### 2.3. Assessment of Adherence

Adherence monitoring to medication treatment was accomplished via a weekly adherence log [22]. Adherence was scored on a scale of 0–4 (0 = not adherent, 1 = 0–25%, 2 = 25–50%, 3 = 50–75%, 4 = 75–100% of prescribed doses taken). Scores were obtained through reports of case managers (who have frequent contact with patients and their families), in discussion with the primary psychiatrist. Patient and family reports were considered in making adherence assessments, as well as reviews of prescriptions and pill counts. Individuals were considered to be adherent if they scored a 4, meaning their compliance was estimated to be between 75–100%. Individuals that were not medication adherent (scores of 0, 1, 2, or 3) based on 6-month adherence measures were not included in the sample (*n* = 36 excluded from 129).

### 2.4. Probable Treatment Resistance Criteria

Probable TR status was investigated at 6 months after entry into the first episode psychosis program based on meeting defined thresholds for symptom change from baseline, in those who were medication adherent. For positive, negative, and total symptom domains, we used 20%, and less than 50% improvement as cut-offs to identify subjects satisfying “probable TR” criteria. Following identifying “probable TR” individuals, individual item scores on the SAPS and SANS (as applicable) were examined at baseline to ensure that individuals meeting criteria met the threshold of at least moderate severity (more than one individual item >2) in terms of symptomology, as suggested by TRIPP ([5]).

### 2.5. Statistical Analysis

All statistical analyses were carried out using SPSS 25 (IBM Corp. Released 2017). Goodness-of-fit tests based on chi-square statistics were performed on the 6 definitions of probable TRS. We tested the observed proportions against the expected proportion of 33% subjects being treatment resistant [1,2], as identified in FES cohorts over 5 years by Lally and colleagues [6]. The definitions that identified the expected proportion of patients were entered as independent predictor variables in 2 separate multiple regression models, with the average of the GAF scores assessed annually over the next 5 years and clozapine use by 5th year being the dependent variables. The definition with the best predictive validity was used to identify ‘probable TR’. Chi-square analyses**/**t-tests were then performed to determine the univariate relationship between baseline variables (independent predictors) and probable TR identified by the extant criteria (dependent variables). For all t-tests, Levine’s test for homogeneity of variances was conducted. For variables showing significant heterogeneity of variance, the corrected *p*-value was used, after adjusting the degree of freedom using a Satterthwaite approximation as implemented in SPSS (v25.0). Logistic regression analyses were then applied to create a model to predict membership within the probable TR group (dependent variable) based on the factors found to be significantly associated in the univariate analysis (independent predictors). Mann-Whitney U tests were used to compare the number of antipsychotic medication trials in individuals with probable and non-probable TR for each symptom improvement threshold. A statistical significance defined by a threshold of *p* < 0.05 was used for all analyses.

## 3. Results

### 3.1. Final Sample

129 patients met criteria for a first episode schizophrenia spectrum disorder and were considered for inclusion in the analysis. Using only individuals that were medication adherent, resulted in a sample size of 93 FES patients (74 male and 19 female), while 36 (30.23%) were not included due to being categorized as non-adherent. One male was missing SANS scores and therefore the total sample size was 92 for analyses assessing negative symptoms. The mean age at onset of psychosis was 24.19. Per diagnostic consensus conference, 70 met criteria for schizophrenia, 20 for schizoaffective disorder, and 2 for schizophreniform disorder. 56 were inpatients at study entry, while 37 were outpatients. The mean chlorpromazine equivalence at 6 months was 227.35 mg. (See Table 1 for further details).

### 3.2. Goodness of Fit for Various Definitions for Probable TR

**Figure 1:**
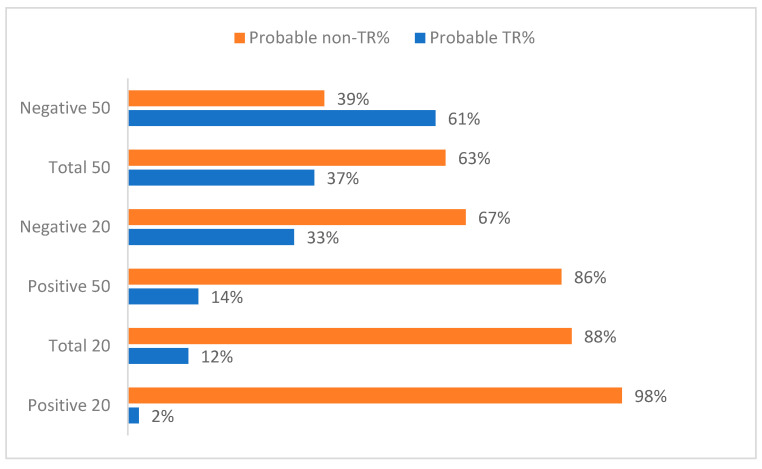
Distribution of probable treatment resistance 6-months after antipsychotic initiation. Across the 3 domains of symptom measures (positive, negative and total), the use of either 20% or 50% cut-off (*y* axis) for defining treatment response by 6 months after induction to a first-episode program results in different proportions of patients being identified as probable cases of treatment resistance (*x*-axis).

We first investigated the prevalence of probable TR at 6 months using 20% and 50% symptom improvement thresholds for positive, negative and total symptoms (see Table 2). Total symptom probable TR < 50% and negative TR < 20% at 6 months resulted in rates closest to those previously described in the literature [1,2] (rates of 37% and 33% respectively). See Table 2 and Figure 1. We further tested the goodness of fit of these models using one-sample chi-square tests with the null hypothesis being that the proportion of TR individuals would be 33% for each criterion. The null hypothesis was rejected for all definitions, with the exception of TR negative <20% (χ2 =0.01, *p* = 0.936, df = 1) and TR total < 50% (χ2 = 0.65, *p* = 0.420, df = 1), meaning the expected frequency of TR for these criteria was approximately 33%. Of those meeting criteria for TR negative < 20%, 77% also met criteria for total symptom TR < 50%, suggesting there was a high degree of overlap between these two categorizations of TR. See Appendix A and Supplementary Tables S1 and S2 for predictors of probable TR based on total <50% criterion.

### 3.3. Ability to Predict Poor Global Functioning over the Next 5 Years

With total symptom improvement <50% and negative symptom improvement <20% at 6 months as independent predictors, multiple regression analysis was conducted to predict the average of 5 annual observations of GAF scores (*n* = 92 for 3 observations; *n* = 82 for year 4; *n* = 72 for year 5; mean imputed for missing values). We tested for multicollinearity using the variance inflation factor (VIF) between the 2 predictors using a threshold of 2, (with a tolerance of less than 0.9 for all predictors) and no evidence of multicollinearity detected. The model was significant (F = 5.52 *p* = 0.006), with the probable TR (defined by total symptoms <50% improvement) being the significant predictor (*t* = −3.15, *p* = 0.002), while 20% negative symptom criteria was not a significant predictor (*t* = 0.94, *p* = 0.35) of the dependent variable GAF at 5 years.

### 3.4. Ability to Predict Clozapine Use by 5 Years

By year 5, 15 out of 92 patients (16.2%) with FES were on clozapine, lower than the expected 33% with TRS. Only 3 out of 15 were receiving it at year 2, indicating that most clozapine initiation occurred between 2 to 5 years of illness. With total symptom improvement <50% and negative symptom improvement <20% at 6 months as independent predictors, multiple regression analysis was conducted to predict the prescription of clozapine at year 5 (dependent variable). The model was not significant (F = 1.45 *p* = 0.24), with both total symptom <50% improvement (*t* = 1.71, *p* = 0.09) and <20% negative symptom improvement not being significant predictors (*t* = −0.92, *p* = 0.36). Following this observation, we undertook a hierarchical regression analysis, wherein <50% total and <20% negative symptom criteria were retained as Block 1 predictors, while <50% positive symptom criterion was entered as Block 2. The R2 of the model increased from 0.03 to 0.11, with the R^2^ change being significant (F = 7.78, *p* = 0.006), with <50% positive symptom improvement at 6 months being a significant predictor of clozapine use at year 5 (*t* = −2.79, *p* = 0.006). The diagnostic odds ratio for the various symptom improvement thresholds at 6 months for future clozapine use is presented in Supplementary Material (Table S3), indicating that the odds of clozapine use at 5th year was highest in those showing <50% positive symptom improvement by 6 months. See Table 3 for a summary of results.

### 3.5. Patterns of Antipsychotic Use

Overall, there were no significant differences in the number of antipsychotic trials for either the <50% or <20% thresholds for either probable or probable non-TR, indicating that the clinician prescribers were not able to foresee the later emergence of TR in this sample. There were no significant differences in the number of antipsychotic medication trials for those with probable and probable non-TR based on total symptom improvement for the thresholds of <20% (U = 853.00, Z = −0.077, *p* = 0.939), or <50% improvement (U = 840.0, Z = −0.187, *p* = 0.852). Similar results of no difference were also observed for positive symptom criteria for the 20% improvement threshold (U = 871.5, Z = −0.657, *p* = 0.511) or the 50% improvement threshold (U= 834.0, Z = −0.803, *p* = 0.422), and negative symptom criteria for 20% improvement threshold (U = 744.0, Z = −1.22, *p* = 0.223) or the 50% threshold (U = 850.0, Z = −0.082, *p* = 0.935). Overall, these results indicate that there were no systematic differences in sequential antipsychotic switching in the first 6 months of entry to the first-episode program among patients with different response trajectories. See Supplementary Table S4 for the range of doses used and different medications prescribed in the 6 months period.

## 4. Discussion

This is the first study to our knowledge to investigate the applicability of response thresholds proposed by TRIPP [5] in a FES sample. In addition, there have been no other studies looking specifically at the importance of considering negative symptom persistence in prospective characterisation of TR in early psychosis. We observe that (1) around 1/3rd of FES subjects show <50% improvement in total symptoms at month 6; (2) this group shows poor global functioning despite treatment over the next 5 years and (3) does not receive more antipsychotic trials than those who show >50% improvement at month 6. We also observe that while <50% improvement in total symptoms at 6 months do not predict later clozapine use, the same threshold when applied for positive symptoms, identifies TR subjects treated with clozapine. These results indicate that identifying a probable TR group in FES at 6 months irrespective of antipsychotic usage data is a viable strategy for prospective and early identification of TRS. These observations inform the timing (6 months), type of measurement (total symptoms) and decision thresholds (<50% improvement) for suspecting TR, when adopting a Measurement Based Care approach in FES clinics.

Our results suggest that at 6 months of treatment, the most ecologically valid definition, based on the expected rate of 33% subjects having a resistant form of schizophrenia, is the failure to improve in total symptoms by 50%, or negative symptoms by 20%. In keeping with our hypothesis, inclusion of negative symptoms in defining TR appears to be crucial to identify the probability of TRS in an acceptable proportion of individuals with FES. Definitions of TR relying solely on positive symptomology were inadequate in identifying cases of probable TR (probable TR rates were 2.2% for the 20% threshold, and 14% for the 50% threshold) likely in keeping with the fact that positive symptom improvement is robust early in the course of schizophrenia [23]. The 20% symptom improvement criteria as suggested by TRIPP [5], seems effective when considering only negative symptom improvement, but fails to identify the expected proportion of patients when positive symptoms are considered in a FES sample. Our results provide preliminary evidence that individuals with probable TR can be identified at as early as 6-months following FES, regardless of the number of antipsychotic trials, and that inclusion of negative symptom improvement is essential in risk-stratification for probable TR in early psychosis.

We have demonstrated the feasibility of prospectively identifying a group of FES subjects that share the risk factors for later TR, even before 2 antipsychotic failures can be observed. Active monitoring for probable TR in early intervention programs can aid in treating these individuals at the earliest possible opportunity, and avoid interventional relapse [24] and subsequent resistance [10]. Clozapine is the only antipsychotic medication with superior efficacy in TR individuals [25] and current guidelines necessitate failure of two antipsychotic agents prior to a trial of clozapine is initiated. The requirement of consecutive antipsychotic trials may contribute to delays in clozapine initiation. Response rates to clozapine following failed antipsychotic trials have been shown to be more robust in patients with FES (75%) [3], relative to chronic samples (40%) [26] and therefore, developing objective clinical indicators for earlier determination of clozapine eligibility is of pivotal importance. As positive symptom improvement is significant at this early stage, focusing exclusively on positive symptom improvement may lead to under-identification of TR individuals, as shown in our work. It is worth noting that despite the uncertainty that surrounds clozapine’s effectiveness for negative symptoms [25], the burden of negative symptoms in early stages of schizophrenia predict later TR (Demjaha et al., 2017) [7].

Limitations: One of the most important limitations is the limited size of our sample; of 129 prospectively followed-up subjects with FES, only 93 had sufficient treatment adherence, with 92 having the requisite clinical follow-up data in the first 6 months (29% attrition). While this reduces the precision of our estimates of probable TR, such attrition is not unexpected in naturalistic clinical settings. While formal assessments of medication adherence were completed, antipsychotic serum levels were not performed. However, our measure of assessing adherence has been validated, and has been shown to be related to pill counts [27]. Symptom data were available at discrete intervals however, the relapse status of individuals between assessments was not recorded for the purposes of this analysis. It is possible that individuals who experienced relapse, particularly prior to 6 months when TR status was assessed, may have been mislabelled as having inadequate response simply because their symptoms were higher at the time of assessment secondary to relapse. However, the fact that TR status at 6 months was associated with impaired symptom improvement during the early months, suggests that for the majority, the development of TR was occurring early on, and was not simply related to an acute symptomatic relapse. Certain service-utilization variables that were not considered in our regression analyses may also affect the probable treatment resistance status, e.g., hospital admission status at baseline. Nevertheless, when we compared the symptom burden at 6 months, we found no significant difference in positive (*t* = 1.2. *p* = 0.21), negative (*t* = 0.72, *p* = 0.4) or total (*t* = 1.1, *p* = 0.27) symptom burden between the patients who were initially hospitalized vs. those who were not. This lack of difference may be due to the relatively shorter duration of hospital admission (PEPP is a community early intervention team that explicitly promotes early discharge) and the lack of hospitalization data during the later course of illness. Finally, our sample only included individuals assessed as being adherent with medication at 6 months, and therefore, our findings relate specifically to the phenomenon of TR in the treatment compliant individuals, and not necessarily TR in the overall schizophrenia population.

## 5. Conclusions

In a naturalistic practice setting, when considering treatment-compliant subjects, the number of AP trials does not differ between probable TR and non-TR. In other words, in the absence of the explicit clinical knowledge of the risk factors, pharmacological practice does not change for patients who are likely to later need clozapine. This is an important aspect to consider in our efforts aimed at mitigating the delay in timely clozapine use. Our results suggest that irrespective of the number of AP trials attempted, if a patient with FES shows less than 50% total symptom burden by 6 months of early intervention, then fast-tracking to clozapine may be warranted. This highlights the need for measurement-based decision-making in FES settings [28].

## Figures and Tables

**Table 1:** Clinical and demographic characteristics of the final sample.

Characteristic	Sample (*n* = 92)
Age	25.72 (7.97)
Gender (M/F)	73/19
Age of onset in years (SD)	24.27 (8.1)
DUP (mean in weeks) (SD)	74.53 (112.5)
DUI (mean in weeks) (SD)	292.3 (275.1)
SAPS baseline (M/SD)	10.5 (3.58)
SANS baseline (M/SD)	12.7 (5.17)
Substance abuse/dependence (Y/N)	23/69
Mode of onset (I/A)	67/23
Family History (Y/N)	32/49

DUP = duration of untreated psychosis. DUI = duration untreated illness. TR = Treatment Resistance. SANS/SAPS = Scale for the Assessment of Negative/Positive Symptoms. Mode of onset (data from 90 subjects)—I = insidious. A = acute. Y = yes. N = no. M = male. F = female. SD = standard deviation.

**Table 2:** Rates of Probable TR and mean number of antipsychotic trials using various criteria.

Domain	Criteria	Probable TR (%)	AP Trials (M/SD)	Non TR (%)	AP Trials
Positive symptoms	<20% <50%	2 (2.2) 13 (14)	2.00/1.41 1.38/0.65	91 (97.8) 80 (86)	1.30/0.50 1.30/0.05
Negative symptoms	<20% <50%	30 (33) 56 (60.8)	1.23/0.50 1.30/0.54	62 (67) 36 (39.13)	1.34/0.54 1.31/0.52
Total symptoms	<20% <50%	11 (12) 34 (36.96)	1.36/0.67 1.32/0.59	81 (88) 58 (63.04)	1.29/0.51 1.29/0.49

AP trials = number of antipsychotic trials; M = mean; SD = standard deviation; TR = treatment resistance. The non TR% are the same as response rates at 6 months time for each domain. For example, 63.04% of treatment-adherent patients showed a 50% or greater reduction in total symptoms, while 86% showed a 50% or greater reduction in positive symptoms domain.

**Table 3:** Summary of results.

Domain	Threshold	Ability to Select a 33% Subgroup by 6 Months of FES	Ability to Predict Low GAF over 5 Years	Ability to Predict Clozapine Use by 5 Years
Positive Symptoms	<20%	NO	-	-
	<50%	NO	-	YES *
Negative Symptoms	<20%	YES	-	-
	<50%	NO	-	-
Total symptoms	<20%	NO	-	-
	<50%	YES	YES	NO

FES = First Episode Schizophrenia; GAF = Global Assessment of Functioning * Based on a R^2^ change of a hierarchical regression analysis with <50% total and <20% negative symptom criteria as Block 1 predictors, and <50% positive symptom criterion as Block 2.

## Data Availability

The data presented in this study are available on request from Lena Palaniyappan (lpalaniy@uwo.ca).

## Supplementary Materials

The following are available online at https://www.mdpi.com/article/10.3390/jpm11080711/s1, Table S1: Factors Associated with Probable TR (<50% improvement on total symptoms).; Table S2: Binary logistic regression analysis of Probable TR total symptom <50% improvement criteria. Table S3: Diagnostic Test Performance of Probable TR using various criteria. Table S4: Details of medications prescribed at baseline and by 6 months.

## Appendix A

**Factors associated with probable TR (total symptoms <50%) at 6 months:** A logistic regression analysis was conducted to predict TR total <50% criteria using all predictors found to be associated with TR in univariate analyses. Patients meeting criteria for probable TR based on having a less than 50% total symptom improvement had worse premorbid functioning (M = 0.37, SD = 0.13) than patients not meeting probable TR on the basis of this criteria (M = 0.28, SD = 0.17) (*t*(80) = −2.526, *p* = 0.014), a longer duration of untreated illness (probable TR: M = 386.21, SD = 340.78; non TR: M = 236.31, SD = 211.28), (*t*(48.37) = −2.31, *p* = 0.025), a lower baseline SAPS score (probable TR: M = 9.35, SD = 3.52; non TR: M = 11.21, SD = 3.47), (*t*(90) = 2.463, *p* = 0.016) and less total symptom percentage improvement over 1 month (probable TR: M = 16.39%, SD = 26.37; non TR: M = 37.38%, SD = 23.46), (*t*(57) = 3.048, *p* = 0.003), and 2 months (probable TR: M = 20.87%, SD = 27.05; non TR: M = 48.70%, SD = 29.83), (*t*(60) = 3.59, *p* = 0.001) (see Supplementary Table S1).

We tested for multicollinearity using the variance inflation factor (VIF) among the predictors using a threshold of 2, (with a tolerance of less than 0.9 for all predictors) with no evidence of multicollinearity detected. A test of the full model was significant, indicating the model as a whole could reliably identify probable TR (total < 50%) (χ2 = 15.90, *p* = 0.007, df = 5). Prediction success overall was 82.2% (96.9% for no probable TR and 46.2% for probable TR), indicating the model was much stronger at ruling out TR. In analyzing the independent predictors in the model (see Supplementary Table S2), none of the variables independently predicted TR status, although total symptom change over 2 months demonstrated trend-level significance (*p* = 0.059).
