# Supplementary material for: Treatment Resistance: A Time-Based Approach for Early Identification in First Episode Psychosis
Source: J Pers Med. 2021 Jul 24;11(8):711. doi: 10.3390/jpm11080711 (PMC8400307; doi:10.3390/jpm11080711)
Supplement: Supplementary file 1 [file jpm-11-00711-s001.zip › jpm-1319009-supplementary.pdf]

Table S1. Factors Associated with Probable TR (<50% improvement on total symptoms).

Characteristic	Probable TR (*n* = 34)	Non TR (*n* = 58)	t/X2	df	sig
PAS (M/SD)	0.37/0.17	0.28/0.16	−2.526	80	0.014 *
DUP (weeks) (M/SD)	104.04/134.98	68.10/105.38	−1.421	90	0.159
DUI (weeks) (M/SD)	386.21/340.78	236.31/211.28	−2.313	48.37	0.025 *
Age of onset (M/SD)	23.96/8.66	24.45/7.80	0.275	90	0.784
SAPS baseline (M/SD)	9.35/3.52	11.21/3.47	2.463	90	0.016 *
SANS baseline (M/SD)	11.38/4.64	13.47/5.35	1.89	90	0.062
Total symp change ratio 1 month	16.39	37.38	2.048	57	0.003 *
Total symp change ratio 2 months	20.87	48.71	3.585	60	0.001 *
Substance abuse/dependence	Y-9; N-25	Y-14; N-44	0.062	1	0.803
Mode of onset	I-23; A-9	I-44; A-14	0.172	1	0.678
Gender	M-28; F-6	M-45; F-13	0.297	1	0.586
Family History	Y-12; N-16	Y-20; N-33	0.201	1	0.654
Number of AP switches (M/SD)	0.2941	0.2759	−0.186	90	0.853
Cpz eq at 6 months (M/SD)	249.51	213.92	−0.884	56.36	0.381

PAS = premorbid adjustment scale. DUP = duration untreated psychosis. DUI = duration untreated illness. TR = Treatment Resistance. SANS/SAPS = Scale for the Assessment of Negative/Positive Symptoms. Total symp change ratio is difference between baseline symptoms and 1, and 2 month symptoms/Baseline symptoms. I = insidious. A = acute. Y = yes. N = no. M = male. F = female. AP = antipsychotic. Cpz eq = chlorpromazine equivalence over 6 months. Note that for all t-tests, Levine's test for homogeneity of variances was conducted. For variables showing significant heterogeneity of variance, the corrected *p*-value was used.

Table S2. Binary logistic regression analysis of Probable TR total symptom <50% improvement criteria.

Predictor	Odds Ratio (95% CI)	*p*-Value
Premorbid adjustment	35.561 (0.168–7520.22)	0.168
Duration of untreated illness	1.002 (0.999–1.005)	0.249
SAPS baseline	1.041 (0.825–1.314)	0.733
Total symp change ratio over 1 month	0.9(0.936–1.016)	0.227
Total symp change ratio over 2 months	0.963 (0.927–1.001)	0.059

SAPS: Scale for the Assessment of Positive Symptoms. Total symptom change ratio = (Baseline total symptoms –N month total symptoms)/Baseline total symptoms. $\chi^2 = 15.90$; $p = 0.007$, Nagelkerke $r^2 = 0.426$; B = −0.901; SE = 0.329; Wald = 7.501; $p = 0.006$; Exp(B) = 0.406.

Table S3. Diagnostic Test Performance of Probable TR using various criteria.

Domain	Threshold	Diagnostic Odds Ratio (DOR)	Log DOR	95% Confidence Intervals	Total Accuracy
Positive Symptoms	<20%	N/A	N/A	N/A	86%
	<50%	6.67	0.824	[−0.47,2.12]	83%
Negative Symptoms	<20%	1.04	0.017	[−1.19, 1.16]	62%
	<50%	0.34	−0.48	[−1.64, 0.69]	39%
Total symptoms	<20%	5.92	0.77	[−0.59,2.13]	83%
	<50%	2.24	0.351	[−0.77,1.47]	64%

N/A = not available.

Table S4. Details of medications prescribed at baseline and by 6 months.

Name of the Antipsychotic Drug	Number at Baseline	Number at 6-Months	Exposure by 6-Months	Dose Range
Chlorpromazine	1	0	1.09%	50 mg/d

Clozapine	0	1	1.09%	375 mg/d
Flupentixol (depot)	1	4	4.35%	20–50 mg/d
Haloperidol (oral)	3	1	3.26%	5–10 mg/d
No antipsychotics	51	0	0.00%	N/A
Olanzapine	15	34	36.96%	5–35 mg/d
Quetiapine	1	6	6.52%	300–550 mg/d
Risperidone	24	43	46.74%	1–8 mg/d
Ziprasidone	0	6	6.52%	40–160 mg/d

The dose range refers to the variation noted across individuals in the recorded dose at the time of assessment. This does not reflect the various doses that were prescribed to an individual when the medications were initiated or titrated before or after the point of assessment.
